# Albuminuria Is Associated with Open-Angle Glaucoma in Nondiabetic Korean Subjects: A Cross-Sectional Study

**DOI:** 10.1371/journal.pone.0168682

**Published:** 2016-12-22

**Authors:** Gyu Ah Kim, Se Hee Park, Jaesang Ko, Si Hyung Lee, Hyoung Won Bae, Gong Je Seong, Chan Yun Kim

**Affiliations:** 1 Institute of Vision Research, Department of Ophthalmology, Severance Hospital, Yonsei University College of Medicine, Seoul, Korea; 2 Division of Endocrinology and Metabolism, Department of Internal Medicine, Yonsei University College of Medicine, Seoul, Korea; 3 Department of Ophthalmology, Eulji University, Daejeon, Republic of Korea; Soochow University Medical College, CHINA

## Abstract

**Objective:**

Systemic vascular dysfunction has been suggested to contribute to glaucomatous damage. Albuminuria is a surrogate marker of endothelial injury, including vessels. However, their relationship is not well understood. This study aimed to investigate the association between albuminuria and the prevalence of open-angle glaucoma (OAG) in nondiabetic subjects.

**Methods:**

We conducted a cross-sectional study of 4186 nondiabetic participants aged 19 years or older from the 2011–2012 Korea National Health and Nutrition Examination Survey. OAG was defined based on the criteria of the International Society for Geographic and Epidemiologic Ophthalmology. Urinary albumin excretion was assessed by the urinary albumin-to-creatinine ratio (UACR). A multivariate logistic regression analysis was performed to evaluate the relationship between albuminuria and OAG.

**Results:**

Among the subjects, 124 had OAG. The weighted prevalences of microalbuminuria (UACR of 30–299 mg/g creatinine [Cr]) and macroalbuminuria (UACR ≥ 300 mg/g Cr) were 3.2 ± 0.3% and 0.4 ± 0.1%, respectively. The percentages of OAG increased in accordance with increasing UACR tertiles. Compared with subjects in the lower UACR tertile, those in the upper tertile showed an increased prevalence of OAG (odds ratio, 1.963; 95% confidence interval 1.072–3.595, *P* = 0.029) after adjusting for demographic factors, laboratory parameters, kidney function, and intraocular pressure. Furthermore, even after excluding 155 subjects with microalbuminuria and 19 subjects with macroalbuminuria, a positive association persisted between the upper UACR tertile (low-grade albuminuria) and an increased prevalence of OAG (odds ratio, 2.170; 95% confidence interval, 1.174–4.010, *P* = 0.014).

**Conclusion:**

Albuminuria, even low-grade, was significantly associated with OAG in nondiabetic subjects. This result implies the role of vascular endothelial dysfunction in the pathogenic mechanism of OAG and suggests that careful monitoring of OAG is required in nondiabetic subjects with albuminuria.

## Introduction

Glaucoma is the leading cause of irreversible blindness and is characterized by a progressive visual field defect with structural damage to the optic disc [1]. According to a recent review, glaucoma was the cause of 10–11% of blindness in Western Europe and North America, and this percentage increased in the last decade [2]. In addition, worsening disease severity was reported to increase the economic burden [3].

The pathogenesis of glaucoma has not been fully revealed. However, elevated intraocular pressure (IOP) and insufficient blood supply to the optic disc are generally accepted as key risk factors. Particularly, systemic vascular factors have been shown to be related to the pathogenesis of open-angle glaucoma (OAG), regardless of whether the diagnosis was normal-tension glaucoma or high-tension OAG [4]. Moreover, the high incidence of systemic cerebrovascular and cardiovascular morbidity and mortality in patients with glaucoma supports this vascular perspective [5–7].

Albuminuria seems to reflect widespread vascular damage that makes an individual susceptible to organ damage [8]. Formerly, it has been described as a marker of diabetic nephropathy [9]. However, recent studies have reported that albuminuria is closely associated with cardiovascular disease in subjects with diabetes, hypertension, and even in the general population [10–12]. Regarding ocular diseases, albuminuria was found to be an independent risk factor for diabetic retinopathy [13] and associated with retinal nerve fiber layer loss suggestive of diabetic optic neuropathy [14] and high IOP [15] in diabetic patients. However, the association between albuminuria and OAG has not been investigated to date.

Therefore, in this study, we aimed to investigate the relationship between albuminuria and OAG in the Korean nondiabetic population using data from the 2011–2012 Korea National Health and Nutrition Examination Survey (KNHANES). Identifying biomarkers that make the optic nerve vulnerable to glaucomatous damage is important to better understand the etiology of and improve screening for this disease.

## Materials and Methods

### Study population

Data from KNHANES V during 2011–2012 were used in this study. The KNHANES, a population-based cross-sectional survey performed by the Korea Centers for Disease Control and Prevention, consists of a health interview survey, nutrition survey, and health examinations. All three parts of the survey were conducted by well-trained examiners in equipped mobile examination centers. Ophthalmologic examinations included visual acuity measurements, IOP measurements, slit-lamp examination, fundus photography, and visual field examination. Fundus photography was taken with a non-mydriatic fundus camera (TRC-NW6s, Topcon, Tokyo, Japan) and a digital camera (Nikon D-80; Nikon, Tokyo, Japan). Visual field tests were performed with frequency doubling technology perimetry (Humphrey Matrix, Carl Zeiss Meditec, Dublin, CA, USA). All participants provided written informed consent. The Institutional Review Board of Severance Hospital approved the study protocol (4-2015-1092) in January 2016.

### Inclusion criteria

Nondiabetic participants aged 19 years or older who underwent both the health interview survey and eye examination were included in the present study. Nondiabetic subjects were defined by participants whose serum fasting glucose levels were lower than 100 mg/dL without a history of a diagnosis of diabetes or previous use of anti-diabetic agents, including insulin.

### Exclusion criteria

Participants were excluded if they had conditions that might influence the visual field test other than glaucoma such as stroke, diabetic retinopathy, and age-related macular degeneration. Individuals showing a shallow peripheral chamber depth lower than 1/4 of the corneal thickness, were excluded to rule out the possibility of angle closure suspect or angle-closure glaucoma. Participants with a previous glaucoma diagnosis or treatment or ocular surgery (glaucoma, cataract, retina, or refractive surgery) were excluded, as those histories might hinder the accurate measurement of subject's original IOP. In addition, glaucoma suspects were excluded (individuals without glaucoma and the presence of one of the following: IOP ≥ 22 mmHg, a vertical or horizontal cup-to-disc ratio ≥ 0.5, violation of the ISNT rule, which means a neuroretinal rim thickness in the inferior > superior > nasal > temporal quadrant order, disc hemorrhage, and retinal nerve fiber layer defects). We also excluded participants with missing values and those with an estimated glomerular filtration rate (eGFR) lower than 60 mL/min/1.73 m^2^, suggesting chronic kidney disease, from this study.

### Laboratory analysis

Blood samples were collected after fasting for at least 8 hours and analyzed for measurements of serum triglyceride, high-density lipoprotein cholesterol, fasting glucose, and creatinine using an automatic analyzer (Hitachi Automatic Analyzer 7600; Hitachi, Tokyo, Japan).

Urinary albumin and creatinine levels were also measured from first morning urine samples using an automatic analyzer (Hitachi Automatic Analyzer 7600; Hitachi, Tokyo, Japan). Moreover, urinary albumin excretion was reported as the urinary albumin-to-creatinine ratio (UACR) in milligrams per gram of creatinine (mg/g Cr), which was calculated as urinary albumin divided by creatinine. For the evaluation of albuminuria, calculating the UACR in untimed spot urine is the preferred method because of its convenience over the collection of timed urine specimens. Numerous studies showed high correlations between UACR in untimed spot urine and albumin excretion rate in timed urine specimens.[16]

The tertile cut-off values were determined by sex separately because men carry greater muscle mass than women, which leads to higher urinary creatinine excretion and relatively lower UACR values. The sex-specific cut-off points for the UACR tertiles were as follows for men and women, respectively: lower tertile, 0.04–1.46 and 0.04–1.91 mg/g Cr; middle tertile, 1.47–3.33 and 1.91–4.77 mg/g Cr; and upper tertile, ≥ 3.34 and ≥ 4.77 mg/g Cr. In addition, to evaluate the relationship between OAG and low-grade albuminuria[17] within the conventionally normal range (UACR < 30 mg/g Cr), additional sex-specific cut-off points for UACR tertiles were set as follows after excluding subjects with microalbuminuria (UACR of 30–299 mg/g Cr) and macroalbuminuria (UACR ≥ 300 mg/g Cr)[18] for men and women, respectively: lower tertile, 0.04–1.41 and 0.04–1.79 mg/g Cr; middle tertile, 1.42–3.13 and 1.79–4.32 mg/g Cr; and upper tertile, 3.14–29.43 and 4.32–29.48 mg/g Cr.

For the assessment of kidney function, eGFR was calculated using the equation from the Chronic Kidney Disease Epidemiology Collaboration (CKD-EPI).[19]

### Definitions

The health interview survey included a standard questionnaire about age, education level, smoking habits, alcohol consumption, exercise, and previous and current medical and comorbid diseases. Heavy alcohol use was defined as drinking ≥ 4 times per week during the year before the interview. Ever-smokers were defined as participants who had ever smoked, regardless of their current smoking status. Moderate exercise was defined as moderate physical activity that causes shortness of breath (for example, swimming, playing tennis, or volleyball) ≥ 5 days per week for ≥ 30 minutes at a time or intense physical activity such as jogging, climbing, cycling, playing soccer, or basketball ≥ 3 days per week for ≥ 20 minutes at a time.

As part of the health examination, waist circumference and systolic and diastolic blood pressures were recorded. Waist circumference was measured to the nearest 0.1 cm in a horizontal plane at the midpoint between the iliac crest and costal margin at the end of normal expiration. Systolic and diastolic blood pressures were measured using a standard mercury sphygmomanometer (Baumanometer, W.A. Baum Co., Copiague, New York, USA) three times, and the average of the second and third measurements was used for the calculation of mean arterial pressure (MAP) as follows: MAP = 1/3 × systolic blood pressure + 2/3 diastolic blood pressure.

### Glaucoma diagnosis

The presence of glaucoma was determined by International Society of Geographic and Epidemiologic Ophthalmology (ISGEO) criteria, as in a previous study [20]. Category 1 criteria require a visual field defect consistent with glaucoma and either a vertical cup-to-disc ratio (VCDR) of at least 0.7 or VCDR asymmetry of at least 0.2 in both eyes. A visual field defect was considered present if at least two different test locations were abnormal. The visual field test was deemed unreliable and repeated if either the fixation error or false-positive rate was greater than 0.33. Category 2 criteria, which are used when visual field results are not definitive or are unavailable, require either a VCDR of at least 0.9 or VCDR asymmetry of at least 0.3 in both eyes. Category 3 criteria, which are used when no information about the visual field or optic disc is available, require a visual acuity less than 3/60 and an IOP higher than 21 mmHg. Individuals with glaucoma and a peripheral chamber depth of ≥ 1/4 of the corneal thickness were considered to have OAG.

### Statistical analysis

The KNHANES participants were not randomly sampled. Thus, a complex sample analysis was used to consider survey sample weights, which were calculated from the sampling rate, response rate, and age/sex proportions of the reference population according to statistical guidance from the Korea Centers for Disease Control and Prevention. To compare weighted demographics among groups according to the presence of OAG and UACR tertile, Student’s *t* test and the chi-squared test were performed. Variables with skewed distributions (UACR and serum triglyceride level) were analyzed using the natural logarithmic (ln) transformation. A multivariate logistic regression analysis was used to evaluate odds ratios for OAG by degree of albuminuria with covariates. These covariates were selected if they showed a significant difference with P<0.1 in baseline characteristics by presence of OAG (age, sex, serum triglyceride level, serum high-density lipoprotein cholesterol level, MAP, education level, ever-smoker status, eGFR) or if they had well-known associations with OAG (IOP). For weighted and adjusted prevalences of OAG with the above-mentioned covariates, an analysis of covariance (ANCOVA) test was used. A test for a linear trend of OAG prevalence across increasing UACR tertiles was conducted by treating the tertiles as a continuous variable. All statistical analyses were conducted using statistical software (SPSS version 20; SPSS, Chicago, IL, USA). *P* values less than 0.05 were considered statistically significant.

## Results

### Demographics of the study population

**Fig 1:**
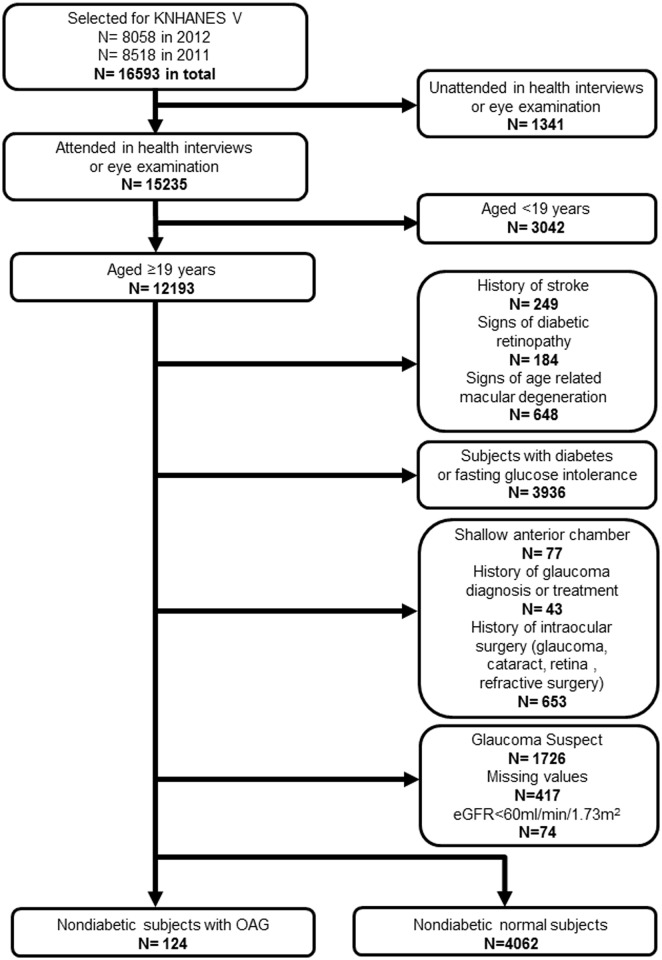
The participation flow chart from the Korea National Health and Nutrition Examination Survey 2011–2012.

The participation flow chart is presented in Fig 1. Among 12193 participants aged 19 years or older who attended both the health interview survey and eye examination from the KNHANES 2011–2012 dataset, 8007 participants (249 subjects with a history of stroke, 184 with diabetic retinopathy, 648 with age-related macular degeneration, 3936 with diabetes or fasting glucose intolerance, 77 with a shallow anterior chamber, 43 with a history of glaucoma diagnosis or treatment, 653 with a history of ocular surgery, 1726 glaucoma suspects, 417 with missing values, and 74 with an eGFR < 60 mL/min/1.73 m^2^) were excluded, and the remaining 4186 participants (124 subjects with OAG and 4062 normal subjects) were analyzed in this study.

The geometric mean of the UACR was 2.4 mg/g Cr in the study population. The weighted prevalences of microalbuminuria and macroalbuminuria were 3.2 ± 0.3% and 0.4 ± 0.1%, respectively. Comparisons of weighted study population demographics according to the presence of OAG and albuminuria tertile are shown in Tables 1 and 2, respectively. The mean age, serum triglyceride level, MAP, UACR, percentage of the male sex, low education level, ever-smokers, and upper UACR tertile were higher in subjects with OAG compared with normal subjects. However, the mean serum high-density lipoprotein cholesterol level and eGFR were lower in subjects with OAG compared with normal subjects. Regarding medical comorbidities, there was no significant difference between the OAG group and the normal group (Table 1). According to albuminuria tertile, mean age, waist circumference, serum triglyceride level, and MAP in the upper tertile were higher than in the lower and middle tertiles. However, the mean serum high-density lipoprotein cholesterol level was lower in the upper tertile compared to the middle tertile. The percentages of subjects with a low education level, hypertension, and renal failure were higher in the upper tertile, whereas the percentage of subjects who performed moderate exercise was higher in the lower tertile. IOP showed no difference among the tertiles (Table 2).

**Table 1:** Demographics of the nondiabetic subjects according to the presence of open-angle glaucoma.

	Normal	OAG	P value
	Unweighted N /Weighted N (4062/14446854)	Unweighted N /Weighted N (124/401779)	
Age (years)	40.3±0.3	49.9±1.8	***<0*.*001***
Male sex (%)	48.8±1.0	65.3±5.1	***0*.*003***
Waist circumference (cm)	79.3±0.2	80.1±0.9	0.443
Triglyceride^a^ (mg/dL)	99.2 (97.0–101.6)	121.3 (104.6–140.7)	***0*.*009***
HDL cholesterol (mg/dL)	50.8±0.2	47.2±1.1	***0*.*001***
Fasting glucose (mg/dL)	88.7±0.1	89.4±0.7	0.307
Mean arterial pressure (mmHg)	87.8±0.2	91.9±1.3	***0*.*002***
Medical comorbidities			
Hypertension (%)	8.8±0.6	13.6±4.4	0.192
Dyslipidemia (%)	5.2±0.4	8.3±3.7	0.313
Renal failure (%)	0.4±0.1	0.8±0.4	0.130
Education level			***0*.*004***
≤Elementary school (%)	11.0±0.7	24.7±4.7	
Middle school graduate (%)	8.0±0.5	7.9±3.2	
High school graduate (%)	44.3±1.1	37.1±5.9	
≥College graduate (%)	36.7±1.0	30.3±5.5	
Heavy drinking (%)	5.6±0.5	10.1±4.2	0.165
Ever smoking (%)	44.6±1.0	66.8±5.7	***<0*.*001***
Moderate exercise (%)	19.6±0.8	16.8±4.3	0.556
eGFR (ml/min/1.73m^2^)	101.1±0.4	96.1±1.8	***0*.*008***
UACR^a^ (mg/g Cr)	2.40 (2.20–2.63)	3.84 (2.47–5.98)	***0*.*041***
UACR tertiles			***0*.*005***
Lower tertile (%)	32.8±1.3	19.9±4.3	
Middle tertile (%)	34.6±1.1	31.2±5.0	
Upper tertile (%)	32.6±1.1	48.9±6.0	
Degree of albuminuria			0.362
Microalbuminuria (%)	3.1±0.3	5.7±2.5	
Macroalbuminuria (%)	0.4±0.1	N/A^b^	
IOP (mmHg)	13.8±0.1	14.4±0.4	0.235

eGFR, estimated glomerular filtration rate; HDL cholesterol, high-density lipoprotein cholesterol; IOP, intraocular pressure; OAG, open-angle glaucoma; UACR, urinary albumin-to-creatinine ratio. All means and frequencies (%) are weighted estimates with standard errors.

^a^ Geometric mean (95% confidence interval)

^b^ Not available due to no subject

**Table 2:** Demographics of the nondiabetic subjects according to albuminuria tertile.

	UACR tertiles			P value	Post hoc analysis
	Lower	Middle	Upper		
UACR range (Male)	0.04–1.46	1.47–3.33	≥3.34		
UACR range (Female)	0.04–1.91	1.91–4.77	≥4.77		
Age (years)	40.1±0.5	39.2±0.4	42.4±0.6	***<0*.*001***	Lower, Middle < Upper
Male sex (%)	49.9±1.7	48.4±1.7	49.4±1.6	0.818	
Waist circumference (cm)	79.2±0.3	78.6±0.3	80.3±0.4	***0*.*002***	Middle < Upper
Triglyceride^a^ (mg/dL)	95.9 (92.4–99.5)	97.5 (93.9–101.2)	106.3 (102.2–110.7)	***0*.*001***	Lower, Middle < Upper
HDL cholesterol (mg/dL)	50.4±0.4	51.5±0.4	50.2±0.4	***0*.*045***	Upper < Middle
Fasting glucose (mg/dL)	88.9±0.2	88.4±0.2	88.9±0.2	0.111	
Mean arterial pressure (mmHg)	86.3±0.4	86.9±0.3	90.5±0.4	***<0*.*001***	Lower, Middle < Upper
Medical comorbidities					
Hypertension (%)	5.6±0.7	6.2±0.7	15.0±1.0	***<0*.*001***	
Dyslipidemia (%)	4.3±0.6	5.1±0.6	6.5±0.7	0.067	
Renal failure (%)	0.1±0.1	N/A^b^	0.6±0.3	***0*.*006***	
Education level				***<0*.*001***	
≤Elementary school (%)	9.7±1.0	8.3±0.8	16.2±1.3		
Middle school graduate (%)	7.7±0.8	7.4±0.9	8.9±0.9		
High school graduate (%)	44.1±1.8	44.5±1.6	43.6±1.8		
≥College graduate (%)	38.5±1.7	39.7±1.6	31.3±1.7		
Heavy drinking (%)	4.2±0.7	6.8±0.9	6.0±0.9	0.097	
Ever smoking (%)	44.5±1.6	44.3±1.7	46.7±1.7	0.495	
Moderate exercise (%)	22.6±1.5	17.5±1.3	18.5±1.3	***0*.*019***	
eGFR (ml/min/1.73m^2^)	100.0±0.5	101.7±0.6	101.2±0.5	***0*.*036***	Lower < Middle
IOP (mmHg)	13.9±0.1	13.8±0.1	13.8±0.1	0.867	

eGFR, estimated glomerular filtration rate; HDL cholesterol, high-density lipoprotein cholesterol; IOP, intraocular pressure; UACR, urinary albumin-to-creatinine ratio. All means and frequencies (%) are weighted estimates with standard errors.

^a^ Geometric mean (95% confidence interval)

^b^ Not available due to no subject

### Weighted and adjusted prevalence of OAG according to albuminuria tertile

**Fig 2:**
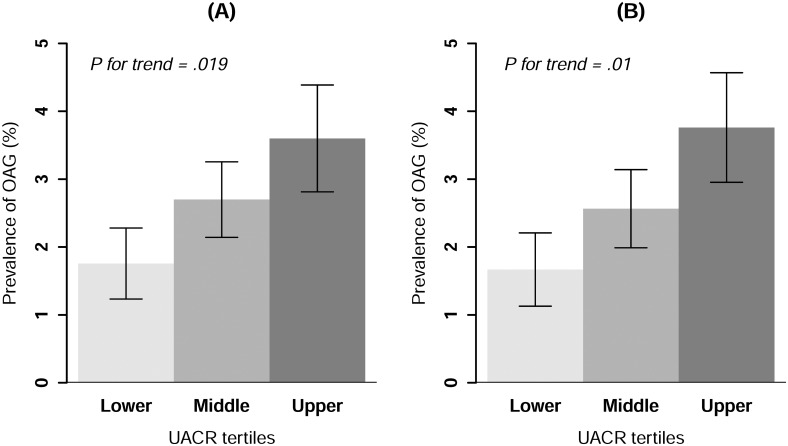
The weighted, multivariable-adjusted prevalence of open-angle glaucoma according to urinary albumin-to-creatinine ratio tertiles in nondiabetic subjects (A) and nondiabetic subjects with low-grade albuminuria within the normal range (B). An analysis of covariance was performed with age, sex, serum triglyceride level, serum high-density lipoprotein cholesterol level, mean arterial pressure, education level, ever-smoker status, estimated glomerular filtration rate, and intraocular pressure as covariates.

After adjusting for age, sex, serum triglyceride level, serum high-density lipoprotein cholesterol level, MAP, education level, ever-smoker status, eGFR, and IOP, the weighted and adjusted prevalences of OAG according to UACR tertile are presented in Fig 2. We found an increasing trend in the weighted and adjusted prevalences of OAG according to albuminuria tertile (1.8 ± 0.5% for the lower tertile, 2.7 ± 0.6% for the middle tertile, and 3.6 ± 0.8% for the upper tertile; *P* for trend = 0.019). This relationship persisted in subjects within the conventionally normal range (UACR< 30 mg/g Cr) after excluding subjects with microalbuminuria and macroalbuminuria (1.7 ± 0.5% for the lower tertile, 2.6 ± 0.6% for the middle tertile, and 3.8 ± 0.8% for the upper tertile; *P* for trend = 0.01).

### Association between OAG and albuminuria according to UACR tertile

Using a multivariate logistic regression analysis, we adjusted for age and sex in model 1, and for age, sex, serum triglyceride level, high-density lipoprotein cholesterol level, MAP, education level, ever-smoker status, eGFR, and IOP in model 2 to obtain odds ratios for the presence of OAG in different UACR tertiles. Setting the lower tertile as the reference, the upper UACR tertile showed a significantly increased odds ratio for the presence of OAG (odds ratio, 2.475; 95% confidence interval, 1.353–4.526, *P* = 0.003). This trend persisted after adjusting for covariates in model 1 (odds ratio, 2.144; 95% confidence interval, 1.160–3.963, *P* = 0.015) and model 2 (odds ratio, 1.963; 95% confidence interval, 1.072–3.595, *P* = 0.029, Table 3). We also conducted an additional matched analysis to minimize the effect of differences in baseline characteristics between the OAG and the normal group. After exact matching for an age and sex on the 1:4 basis, 124 subjects with OAG and 496 normal subjects were included and no statistically significant difference was found between groups in the mean age (49.9±1.8 in OAG group, 50.2±0.8 in normal group) and sex composition (65.3±5.1% of male in OAG group, 65.0±2.4% of male in normal group). Using the same logistic regression model above mentioned, the association between OAG and albuminuria remained statistically significant in the matched cohort as well (Table 4). Furthermore, even after excluding subjects with microalbuminuria and macroalbuminuria, the upper UACR tertile (low-grade albuminuria within the normal range) also showed a significantly increased odds ratio for the prevalence of OAG in models 1 and 2 (model 2: odds ratio, 2.170; 95% confidence interval, 1.174–4.010, *P* = 0.014, Table 5).

**Table 3:** Odds ratios for the presence of open-angle glaucoma among different urinary albumin-to-creatinine ratio tertiles in nondiabetic subjects.

					Model 1		Model 2	
UACR tertile	UACR range (Male)	UACR range (Female)	unadjusted OR	p-value	adjusted OR	p-value	adjusted OR	p-value
**Lower**	0.04–1.46	0.04–1.91	1 [Ref]		1 [Ref]		1 [Ref]	
**Middle**	1.47–3.33	1.91–4.77	1.489 (0.828–2.676)	0.183	1.538 (0.857–2.761)	0.148	1.530 (0.843–2.777)	0.161
**Upper**	≥3.34	≥4.77	2.475 (1.353–4.526)	***0*.*003***	2.144 (1.160–3.963)	***0*.*015***	1.963 (1.072–3.595)	***0*.*029***

OR, odds ratio; UACR, urinary albumin-to-creatinine ratio. Model 1 was adjusted for age and sex. Model 2 was adjusted for age, sex, serum triglyceride level, serum high-density lipoprotein cholesterol level, mean arterial pressure, education level, whether the subject had ever smoked (yes/no), estimated glomerular filtration rate, and intraocular pressure.

**Table 4:** Odds ratios for the presence of open-angle glaucoma among different urinary albumin-to-creatinine ratio tertiles in nondiabetic subjects in the matched cohort.

					Model 1		Model 2	
UACR tertile	UACR range (Male)	UACR range (Female)	unadjusted OR	p-value	adjusted OR	p-value	adjusted OR	p-value
**Lower**	0.05–1.63	0.05–2.06	1 [Ref]		1 [Ref]		1 [Ref]	
**Middle**	1.64–3.79	2.09–6.07	1.599 (0.859–2.975)	0.886	1.589 (0.852–2.965)	0.949	1.587 (0.82–3.073)	0.874
**Upper**	≥3.81	≥6.08	2.372 (1.235–4.554)	***0*.*023***	2.440 (1.253–4.753)	***0*.*022***	2.302 (1.154–4.595)	***0*.*042***

OR, odds ratio; UACR, urinary albumin-to-creatinine ratio. Model 1 was adjusted for age and sex. Model 2 was adjusted for age, sex, serum triglyceride level, serum high-density lipoprotein cholesterol level, mean arterial pressure, education level, whether the subject had ever smoked (yes/no), estimated glomerular filtration rate, and intraocular pressure.

**Table 5:** Odds ratios for the presence of open-angle glaucoma among different urinary albumin-to-creatinine ratio tertiles in nondiabetic subjects with low-grade albuminuria within the normal range.

					Model 1		Model 2	
UACR tertile	UACR range (male)	UACR range (Female)	unadjusted OR	p-value	adjusted OR	p-value	adjusted OR	p-value
**Lower**	0.04–1.41	0.04–1.79	1 [Ref]		1 [Ref]		1 [Ref]	
**Middle**	1.42–3.13	1.79–4.32	1.439 (0.793–2.613)	0.231	1.511 (0.834–2.737)	0.172	1.517 (0.834–2.759)	0.172
**Upper**	3.14–29.43	4.32–29.48	2.472 (1.324–4.615)	***0*.*005***	2.279 (1.215–4.273)	***0*.*010***	2.170 (1.174–4.010)	***0*.*014***

OR, odds ratio; UACR, urinary albumin-to-creatinine ratio. Model 1 was adjusted for age and sex. Model 2 was adjusted for age, sex, serum triglyceride level, serum high-density lipoprotein cholesterol level, mean arterial pressure, education level, whether the subject had ever smoked (yes/no), estimated glomerular filtration rate, and intraocular pressure.

## Discussion

This is the first cross-sectional study to demonstrate an association between albuminuria and OAG in nondiabetic individuals in Korea. Since, unlike OAG, diabetic neovascular glaucoma occurs secondary to obstruction of the trabecular meshwork by neovascular membrane that develops in response to retinal ischemia, we confined the study population to nondiabetic subjects. The weighted prevalences of microalbuminuria and macroalbuminuria in this study were found to be lower than those previously reported (5.2% for microalbuminuria and 1.0% for macroalbuminuria), as the previous study did not exclude diabetic patients [21]. The percentages of OAG increased in accordance with increasing UACR tertiles. Moreover, the upper albuminuria tertile showed a significantly increased risk of OAG compared to the lower tertile. Interestingly, these relationships persisted in subjects with low-grade albuminuria below the conventional threshold for microalbuminuria.

Several studies investigated the relationship between urinary albumin excretion and ocular factors in non-glaucomatous patients with type 2 diabetes. According to these studies, urinary albumin excretion was associated with a high IOP [15] and retinal nerve fiber layer defects [14], which were believed to have close relationships with glaucoma. While earlier studies focused on the relationships between urinary albumin excretion and diabetic optic neuropathy or ocular hypertension, we focused on the influence of albuminuria on OAG by excluding patients with diabetes and impaired glucose tolerance. In the present study, IOP did not show a significant difference among the different albuminuria tertiles, contrary to results of Choi et al [15]. We speculate that this discrepancy might result from the different study designs. In other words, we excluded non-glaucomatous subjects with IOPs higher than 21 mmHg as glaucoma suspects, and excluded subjects with diabetic nephropathy who might have high enough UACR levels to influence IOP. Moreover, we treated IOP as a continuous variable, whereas Choi et al [15] treated IOPs as categorical variables sorted into categories of high IOP (IOP ≥ 18 mmHg) or not. On the other hand, the higher prevalence of OAG in the upper UACR tertile despite similar IOPs may imply other pathogenic factors for OAG besides elevated IOP.

Recently, albuminuria has emerged as an independent predictor for cardiovascular events such stroke, myocardial infarction, and death both in diabetic and nondiabetic individuals [10,11]. The precise mechanism underlying the link between albuminuria and cardiovascular events is uncertain. However, it has been speculated that a glomerular albumin leak reflects widespread atherosclerosis-mediated capillary vasculopathy and endothelial dysfunction, which impair endothelium-dependent vasodilation [22]. And then, a reduced arterial dilatory response is considered to contribute to an increased cardiovascular risk [23].

Insufficient ocular blood supply has been proposed as one of the contributing factors in the pathogenesis of glaucoma [24]. Ocular blood flow could be influenced and compromised by various systemic factors such as vascular endothelial dysfunction [25–27], blood pressure behavior [28,29], and autonomic dysfunction [30] in patients with glaucoma. Of those, vascular endothelial dysfunction in glaucoma was demonstrated by reduced brachial artery flow-mediated vasodilation [25,27] or exaggerated vasoconstriction of arteries dissected from gluteal fat biopsies in response to 5-hydroxytryptamine and endothelin-1 [26]. Taken together, we assumed that a common angiopathic mechanism contributed to the link between albuminuria and OAG. In addition, it is noteworthy that the association between albuminuria and OAG via an angiopathic mechanism became apparent at low-grade albuminuria levels, as early as the association between albuminuria and cardiovascular disease appeared in the general population [31,32]. Although the association between albuminuria and OAG might be confounded by hypertensive chronic kidney disease, we could infer that impaired renal function had little influence on our result considering that the association remained statistically significant even for subjects within the normal range of albuminuria.

There are several limitations to this study. First, because of its cross-sectional nature, we are unable to confirm a causal relationship between albuminuria and OAG. Second, the use of an angiotensin receptor blocker or angiotensin converting enzyme inhibitor that might reduce the level of albuminuria or statin therapy that might ameliorate endothelial function [33] could not be taken into account because of a lack of detailed information regarding medication in the KNHANES dataset. Third, as normal-tension glaucoma comprises the distinct majority of OAG cases in Korea [34], the relationships between albuminuria and OAG in other races with a low proportion of normal-tension glaucoma in OAG should be further investigated.

In conclusion, this is the first nationwide study to show a significant association between albuminuria and OAG in nondiabetic subjects. This result supports the vascular perspective in the pathogenesis of OAG. In addition, careful monitoring for glaucomatous damage may be needed in nondiabetic subjects with albuminuria, and even in those with low-grade albuminuria within the conventionally normal range.
